# Parenteral Antioxidant Supplementation at Birth Improves the Response to Intranasal Vaccination in Newborn Dairy Calves

**DOI:** 10.3390/antiox10121979

**Published:** 2021-12-12

**Authors:** Arpita Nayak, Angel Abuelo

**Affiliations:** Department of Large Animal Clinical Sciences, College of Veterinary Medicine, Michigan State University, 736 Wilson Rd., East Lansing, MI 48824, USA; nayarkar1@msu.edu

**Keywords:** dairy cattle, micronutrients, mucosal immunity, oxidative stress

## Abstract

Newborn calves experience oxidative stress throughout the first month of their life, which is known to decrease lymphocyte functions relevant to vaccine responsiveness. Thus, this study aimed to determine the extent to which parenteral antioxidant supplementation given at birth improves the response to an intranasal viral vaccine in the first month of life of newborn dairy calves. For this, 21 calves were randomly assigned at birth to one of two commercially available antioxidant micronutrient supplements or a placebo group receiving 0.9% sterile saline (*n* = 7/group). Serum and nasal secretion samples were collected before administration of treatments and an intranasal vaccine against respiratory viruses (bovine herpesvirus type 1, bovine syncytial respiratory virus, and parainfluenza 3), and once weekly for the first four weeks of age. Systemic redox balance was determined in serum. Immunoglobulin A specific for bovine herpesvirus 1 and bovine syncytial respiratory virus was quantified in nasal secretions as a proxy to intranasal vaccine responsiveness. Our results showed that parenteral administration of antioxidants at birth improved calves’ redox balance. Additionally, calves receiving antioxidant supplementation had higher concentrations of immunoglobulin A in their nasal secretions than calves in the control group. Thus, we conclude that supplementation of calves with antioxidants at birth could be a practical strategy to improve intranasal vaccine response. Future larger studies should evaluate the extent to which this increased mucosal response to intranasal vaccination could result in decreased calf morbidity and mortality.

## 1. Introduction

Neonatal calf morbidity and mortality have been reported at elevated risks in several countries, including the USA, Canada, and Australia [1,2,3]. Infectious diseases such as diarrhea or respiratory disease are the major causes of disease and mortality during the first months of life [4]. A major contributing factor to this high disease incidence in the neonatal stage is the inability of calves to mount an effective immune response [5].

Intranasal vaccination of calves against respiratory viruses at birth has become a common practice to circumvent inference of passive immunity through the stimulation of mucosa-associated lymphoid tissue [6]. This results in the production of immunoglobulin A (IgA) in mucosal surfaces [7], which provides the first line of defense against respiratory pathogens [8]. Nevertheless, the immune response elicited by vaccination in newborn calves is lower than in adult cattle [5]. Newborn calves experience oxidative stress throughout the first months of life [9,10,11], which reduces immune cell functions relevant to vaccination responsiveness [12].

Parenteral supplements containing micronutrients with antioxidant capabilities are licensed in the USA for administration to cattle and could be used to assuage oxidative stress in newborn calves. To the best of our knowledge, however, the effect of improving antioxidant capacity on the calves’ response to intranasal vaccination has hitherto not been investigated. We hypothesized that calves receiving a parenteral antioxidant supplement would have an improved redox balance, resulting in a greater response to intranasal vaccination. Thus, we aimed to investigate the extent to which the two commercially available parenteral antioxidant supplements could change the systemic redox balance and nasal secretion antigen-specific IgA concentrations in newborn dairy calves throughout their first month of life.

## 2. Materials and Methods

### 2.1. Animals, Treatment Allocation, and Sample Collection

For this study, we used 21 Holstein calves from a commercial farm associated with the Michigan State University Training Center for Dairy Professionals (Elsie, MI, USA). Calves were enrolled at birth. After calving, calves were left with their dams in the individual calving pen for 30 min. Subsequently, calves were weighed; had their navels dipped in an iodine solution; received 4 L of > 22% Brix warm thawed colostrum via orogastric tube, a commercial antibody oral supplement against enteric pathogens (First Defense; Immucell Corporation, Portland, ME, USA), and a commercial live intranasal vaccine (Bovilis Nasalgen^®^ 3, Merck Animal Health, Madison, NJ, USA) against the respiratory viruses parainfluenza 3, bovine herpesvirus type 1, and bovine respiratory syncytial virus; and received the study treatments. Calves received another 2 L of colostrum at approximately 6 h of life. Calves were housed in individual in-house stalls and fed 3 L of milk replacer (Cow’s Match, Land O’Lakes Inc., Arden Hills, MN, USA) at 14% solids, 3 times/day throughout the first month of life. Water was available ad libitum from the first day, and starter concentrate was offered starting at week 1 of age. 

Calves were allocated to 1 of the 3 treatment groups (*n* = 7/group) using randomization software (www.graphpad.com/quickcalcs/randomSelect1/; last accessed 12 May 2021), stratifying by sex. The treatment groups consisted of the two parenteral antioxidant micronutrient supplements commercially available in the USA and a placebo control group. Supplement A (Bo-Se^®^, Merck Animal Health, Madison, NJ, USA) contained selenium (2.19 mg/mL sodium selenite) and Vitamin E (50 mg/mL *D*-alpha tocopheryl acetate), whereas Supplement B (Multimin^®^ 90, Multimin North America Inc., Fort Collins, CO, USA) contained zinc (60 mg/mL), copper (15 mg/mL), selenium (5 mg/mL), and manganese (10 mg/mL). Supplements were administered subcutaneously at their labeled dosage of 0.07 mL/kg for Supplement A, and 0.022 mL/kg for Supplement B. Calves in the placebo group received 3 mL of sterile 0.09% saline subcutaneously. Farm staff but not researchers were blinded to treatment allocation. Two calves of the study died between week 1 and week 2 samplings, and the samples collected prior to death were retained in the study as calves were clinically healthy when samples were collected.

Serum and nasal secretion samples were collected from calves at birth (prior to the administration of colostrum, treatments, and vaccines) and weekly thereafter during the first month of life (median (range) age at sampling: 7 (6–8), 14 (13–15), 21 (20–22), and 30 (29–31) days). For serum collection, blood was collected via jugular venipuncture using 10 mL vacuumed tubes without anticoagulant (Trace Element Serum; Becton Dickinson, Franklin Lakes, NJ, USA). Tubes were allowed to clot for 15 min and centrifuged at 2000× *g* for 15 min. The serum was then harvested, aliquoted into 1.5 mL cryovials, flash-frozen in liquid nitrogen, and stored at −80 °C pending analyses within 1.5 months of collection. Nasal secretions were collected as previously described with minor modifications [13,14]. Briefly, a sterilized foam plug was inserted into one nostril of each calf for up to 5 min or until the foam plug was saturated. Once saturated, the foam plug was removed from the calf’s nostril and inserted into a 5 mL syringe with the plunger removed. The plunger was then reinserted into the syringe to plunge the nasal secretion sample out of the foam plug and the syringe into two 1.5 mL cryovials. These vials were flash-frozen in liquid nitrogen and stored at −80 °C until analysis within 1.5 months of collection.

Nasal secretions were also collected following the same protocol from 5 clinically healthy mid-lactation (210–230 days in milk) Holstein cows in second to fourth lactation housed in the Michigan State University Dairy Cattle Teaching and Research Center (East Lansing, MI, USA). These samples were collected immediately before and 5 days after administering the same intranasal vaccine that calves received (Bovilis Nasalgen 3, Merck Animal Health, Madison, NJ, USA). The samples from each time point were pooled together to serve as negative and positive reference samples, respectively, for assay quality-control purposes to ensure that the ELISA assays could accurately detect low and high antigen-specific immunoglobulin A concentrations. Mid-lactation cows were selected because they are expected to have a robust immune response [15].

### 2.2. Assessment of Transfer of Passive Immunity

To assess passive immunity transfer from the dam to the calf via colostrum, the concentration of serum total protein was estimated in the sample collected at week 1 of age using a digital refractometer (MISCO, Solon, OH, USA). This method has a high accuracy for identifying animals with failed passive immunity transfer [16] and is routinely used in dairy herds [17]. The refractometer was zeroed with distilled water prior to each determination.

### 2.3. Systemic Redox Balance Determination

Redox balance was assessed in serum as previously described [18]. A commercially available fluorometric assay (OxiSelect In Vitro ROS/RNS Assay Kit; Cell Biolabs Inc., San Diego, CA, USA) was used to measure reactive oxygen and nitrogen species (RONS) in serum as a marker of oxidant production. In brief, a dichlorofluorescent dye was added to and reacted with free radicals within the sample, yielding a fluorescent product. Thus, the fluorescent intensity is proportional to the total RONS content in the sample. Fluorescence was determined at 480 nm of excitation and 530 nm of emission using a Synergy H1 Hybrid plate reader (Biotek, Winooski, VT, USA). A standard dichlorofluorescent dye curve was included to ensure that the dye could be detected at various concentrations. Samples were analyzed in duplicate, and those with a coefficient of variation (CV) ≥ 10% were rerun. Values of RONS are presented as relative fluorescent units.

The serum’s antioxidant potential (AOP) was measured using the Trolox equivalent antioxidant capacity, as previously described [19]. Briefly, the AOP of a sample was expressed as the equivalence of a known Trolox (synthetic vitamin E analog) standard (Sigma-Aldrich, St. Louis, MO, USA) concentration, resulting in a similar reduction of the generated radical 2,2′-azino-bis-3-ethylbenzothiazoline-6-sulfonic acid (Sigma, St. Louis, MO, USA) determined using the standard curve. Samples were analyzed in triplicate, and those with a CV ≥ 10% were rerun.

Redox balance was assessed as the ratio of pro-oxidant to total antioxidant defenses (RONS/AOP), namely the oxidant status index (OSi), as it accurately detects changes in redox balance during the transition period of dairy cattle [20]. An increase in the ratio suggests a higher risk for oxidative stress due to increased pro-oxidant production or defensive antioxidant depletion.

### 2.4. Quantification of Mucosal Antigen-Specific Immunoglobulin A

Nasal secretion samples were analyzed for BHV1 and BRSV-specific IgA using an ELISA assay as previously described [13,14]. Briefly, BHV1 and BRSV stock solutions (National Veterinary Services Laboratory, US Department of Agriculture, Ames, IA, USA) were inactivated with UV light for 20 min inside a biosafety cabinet. The BHV1 virus stock solution had a concentration of 13,600 TCID_50_/μL and was diluted 1:100 with coating buffer (Thermo Fisher Scientific, Waltham, MA, USA). In contrast, the BRSV stock solution concentration was 600 TCID_50_/μL and was diluted 1:50 with the same coating buffer. All ELISA plates were prepared at the same time. For this, 96-well plates were coated with diluted solutions and incubated overnight at 4 °C. After incubation, plates were washed three times with 1X ELISA wash buffer (Thermo Fisher Scientific, Waltham, MA, USA). Then, plates were blocked and incubated at room temperature for one hour with ELISA Blocker Blocking Buffer (Thermo Fisher Scientific, Waltham, MA, USA) to prevent non-specific binding of antibodies. Subsequently, the blocking buffer was removed, and the plates were left to dry uncovered inside a biosafety cabinet for 2 h at room temperature. Once dry, the plates were sealed and stored with a desiccant at −20 °C until analysis. The detection antibody was also prepared ahead of analysis. An anti-bovine IgA HRP-conjugated sheep polyclonal antibody (Bethyl Laboratories Inc., Montgomery, TX, USA) was used as detection antibody and was diluted 1:50,000 with 1X PBS containing 0.2% bovine serum albumin. The detection antibody solution was stored at 4 °C until analysis. All analyses were completed within 1 week after preparation.

Nasal secretion samples were thawed on ice for 30 min and diluted 1:10 with 1X PBS containing 0.1% Tween^®^20 (Thermo Fisher Scientific, Waltham, MA, USA). All samples were analyzed in duplicate. Blanks and negative and positive reference samples were included in duplicate in each plate. Prior to analysis, coated plates were removed from −20 °C storage and washed three times with 1X ELISA wash buffer. Diluted nasal secretions (100 μL) were pipetted into each well, and plates were sealed and incubated at room temperature for 1 h. Subsequently, plates were washed 4 times with 1X ELISA wash buffer, 100 μL of detection antibody solution was added to each well, and plates were sealed and incubated at room temperature for 1 h. Following incubation, plates were rewashed 4 times with 1X ELISA wash buffer, 100 μL of TMB Stabilized Chromogen (Thermo Fisher Scientific, Waltham, MA, USA) was added, and plates were incubated at room temperature for 20 min. After incubation, 100 μL of stop solution (Thermo Fisher Scientific, Waltham, MA, USA) was added to all wells, and optical density at 450 nm (OD_450_) was determined immediately in a Synergy H1 Hybrid plate reader (Biotek, Winooski, VT, USA). Thus, OD_450_ readings are proportional to the concentration of IgA present in the nasal secretion samples. The ELISA cut-off was calculated as the mean + 3 standard deviations of the OD_450_ reading of the negative control samples.

### 2.5. Statistical Analyses

Statistical analyses were conducted using JMP Pro 15 (SAS Institute, Cary, NC, USA). Kruskal–Wallis test was used to compare birth weights and the concentration of serum total protein at week 1 of age among treatment groups. Sex distribution across groups was compared with Fisher’s exact test. Mixed models with repeated measures were built for each of the outcome variables of the study (RONS, AOP, OSi, BHV1-IgA, and BSRV-IgA). Treatment (supplement A, supplement B, or control), time (week 0, 1, 2, 3, or 4 of age), and their interaction were the main effects. Sex was included as a random effect. Five covariance structures were tested (unstructured, autoregressive 1, variance components, compound symmetry, and Toeplitz), and the one resulting in the lowest Akaike information criterion was chosen. The degrees of freedom were approximated with the Kenward–Roger method. Model assumptions were assessed by evaluating the homoscedasticity and normality of residuals. To ensure homoscedasticity of residuals, the data of BHV1-IgA and BSRV-IgA were log-transformed. Data are presented as least squares means (95% confidence intervals). Tukey’s honest significance test was used for post hoc pairwise comparisons. Statistical significance was declared at *p* < 0.05.

## 3. Results and Discussion

### 3.1. Homogeneity of Groups

By design, sex was equally distributed among treatment groups. Furthermore, there were no differences among groups in birth body weight or serum total protein concentrations at week 1 of age (Table 1). All study calves had serum total protein concentrations higher than 6.2 g/dL, indicating that all calves had an excellent transfer of passive immunity according to the current standards [21]. Calves with low passive immunity transfer have a greater response to vaccination early in life than those with good passive immunity, attributable to lower interference of maternal antibodies [6]. Therefore, differences in vaccination responsiveness among groups in this study cannot be attributed to different degrees of passive immunity.

### 3.2. Redox Balance

Calves receiving supplement A or B showed lower serum RONS concentrations, higher serum AOP, and lower OSi values than calves in the control group (Table 2), thus indicating that the parenteral antioxidant supplementation given at birth resulted in an improved systemic redox balance in the supplemented calves. 

**Figure 1:**
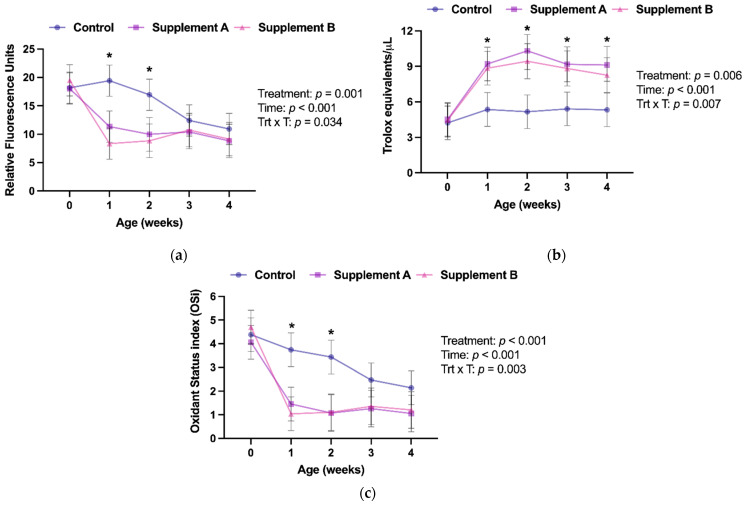
Changes in (**a**) Reactive Oxygen and Nitrogen Species, (**b**) Antioxidant Potential, and (**c**) Oxidant Status index (arbitrary units) throughout the first 4 weeks of age. Results were analyzed using mixed models with repeated measures, including the main effects of treatment (control, supplement A, or supplement B), time (weeks of age), and the treatment x time interaction (Trt x T). Week 0 samples were collected prior to colostrum ingestion or administration of treatments. Results are presented as least squares means and 95% confidence intervals. * denotes differences (*p* < 0.05) between the control and the supplement groups at a given age point as reported by Tukey’s honest significance test.

Examining changes over time, supplements A and B were effective in improving AOP throughout the first 4 weeks of life compared to the control placebo group (Figure 1b). However, this improved AOP only resulted in lower RONS serum concentrations for the first 2 weeks (Figure 1a). Consequently, the redox status of the calves, as assessed by OSi, between supplemented and placebo control calves was only statistically different in weeks 1 and 2 (Figure 1c). 

Newborn calves have been reported to experience oxidative stress throughout the first month of life [10,11], which has been documented to negatively affect the responsiveness of their immune system [12,22]. This study aimed to investigate to which extent supplementing antioxidants to newborn calves using commercially available micronutrient parenteral supplements improves their redox status and immune response to intranasal vaccination. As undertaken in the study, supplementation of either commercial product successfully improved the redox balance of calves during the first 2 weeks of life.

Interestingly, despite improved AOP throughout the study period, supplemented calves only showed improved redox balance, as assessed by OSi, during the first 2 weeks of life. This is attributed to a decrease in the pro-oxidant load of control calves in weeks 3 and 4 of age, as indicated by lower RONS concentrations at these time points than in week 2 (*p =* 0.061 and *p =* 0.017, respectively), as the AOP of control calves remained unchanged throughout the study. The literature on the redox biology of neonatal dairy calves is scarce, and findings from mature cattle cannot be directly translated to newborns [23]. However, a previous study found no changes in serum reactive oxygen species in the first month of life [9], which contradicts our findings. However, pro-oxidant load in calves is thought to be associated with feed consumption, metabolic activity, and growth rate [24]. Thus, the differences in liquid feeding between the previous study (approx. 800 g milk replacer/day) and the current study (approx. 1260 g/day) might explain the differences between the studies. Another factor to consider is the method used to estimate AOP in this study. The different analytical methods available have advantages and disadvantages [25]. One of the limitations of the Trolox equivalent antioxidant capacity method used is that in addition to the low molecular weight substances with antioxidant activity such as those contained in the supplements, it also measures albumin, which represents about 50% of the final value in human samples [26]. However, AOP remained stable in the control group throughout the study period, and previous reports documented no changes in serum albumin concentrations during the first 3 months of life of calves [23]. Thus, although we did not measure albumin in the calves, we believe that the documented changes in redox balance are not influenced by potential age-dependent changes in serum albumin concentrations.

Lastly, there were no statistical differences between both supplements in any of the variables used to assess redox balance (Table 2). Both supplements are licensed for use in cattle, and previous studies have documented their use in newborn calves [27,28]. Although these studies attempted to characterize the effect of their use in calves’ redox balance, they employed methods currently considered unreliable to assess systemic redox status, such as measuring a small set of individual antioxidants [29,30], preventing direct comparison with our results. We observed a similar effect of both supplements on AOP despite Supplement A containing vitamin E and selenium and Supplement B being a combination of trace minerals with antioxidant properties (Zn, Se, Cu, and Mn). However, our study was not designed to compare the effectiveness of both products as this was not an objective. Given that this study is underpowered to compare the commercially available products, future studies should evaluate the superiority or non-inferiority of both supplements to provide evidence-based antioxidant supplementation guidelines to dairy producers.

### 3.3. Intranasal Vaccine Response

The response to an intranasal vaccine was assessed by measuring nasal secretion IgA against two of the viral antigens contained in the commercial trivalent vaccine. Intranasal vaccines are routinely used on farms in newborn calves to circumvent interference of passive immunity with parenteral vaccines [6]. These vaccines stimulate IgA production in mucosal surfaces [7], and the presence of anti-BHV1 and anti-BRSV IgA in the mucosa of the upper respiratory tract has been associated with protecting calves against BHV1 and BRSV infections, respectively [31,32]. Thus, the antigen-specific IgA response in mucosal secretions is routinely used to assess the response to vaccines administered intranasally [13,14,33].

**Figure 2:**
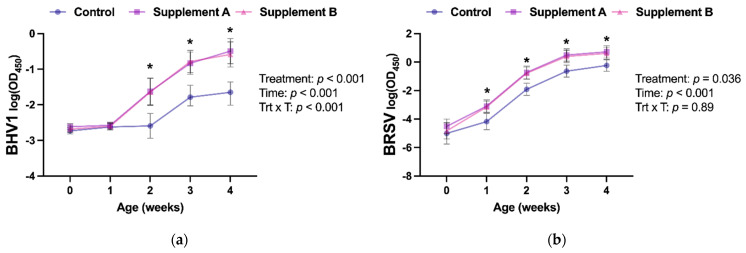
Immunoglobulin A responses in nasal secretions throughout the first 4 weeks of age against (**a**) Bovine Herpesvirus-1 (BHV1) and (**b**) Bovine Respiratory Syncytial Virus (BRSV). Antigen-specific immunoglobulin A was measured using an ELISA assay, and results were analyzed using mixed models with repeated measures, including the main effects of treatment (control, supplement A, or supplement B), time (weeks of age), and the treatment x time interaction (Trt x T). Week 0 samples were collected prior to administration of a commercial intranasal vaccine. Results are presented as least squares means and 95% confidence intervals of the logarithm of the optical density at 450nm (OD_450_). OD_450_ readings are proportional to the amount of immunoglobulin A in the sample. * denotes differences (*p* < 0.05) between the control and the supplement groups at a given age point as reported by Tukey’s honest significance test.

In this study, we investigated the effect of improving the antioxidant capacity of newborn calves on their response to intranasal vaccination. Prior to vaccine and colostrum administration at birth, calves in all groups had similar anti-BHV1 and anti-BSRV IgA responses in their nasal secretions (Figure 2a,b), with values below the ELISA cut-offs (log(OD_450_) = − 1.15 and − 2.03 for BHV1 and BRSV, respectively). A significant treatment effect was identified for anti-BHV1 and -BSRV IgA (*p* < 0.001 and *p* = 0.036, respectively), with increased anti-BHV1 IgA responses in the calves receiving either antioxidant supplement starting at week 2 of age. In contrast, an increased response in anti-BRSV IgA in supplemented calves was already detected at week 1 of age. Interestingly, the anti-BHV1 IgA response in nasal secretions in the control calves was similar between weeks 1 and 2 (*p* = 0.97), whereas calves receiving an antioxidant supplementation showed statistically higher responses between the same timepoints (*p* < 0.0006). Similarly, control calves had similar anti-BRSV IgA responses between birth and week 1 (*p* = 0.87), whereas supplemented calves already exhibited increased IgA at week 1 compared to the basal sampling at birth (*p* < 0.02), thus indicating that the administration of an antioxidant supplement at birth resulted in a faster and greater response to the BHV1 and BRSV components of the vaccine. No differences in their capacity to improve vaccination response were detected between commercial antioxidant supplements (Table 2).

Previous studies in calves have documented improved immune responses to vaccines delivered parenterally when antioxidant supplements were administered at the time of vaccination [34]. However, in addition to using a different route of administration of the vaccine, calves were vaccinated at an older age (3.5 months) when calves are known to have a mature immune system [5]. Nevertheless, these studies demonstrated the ability of parenteral antioxidant supplementation to improve immune responses. To the best of our knowledge, however, this is the first study that reports improved responses to intranasal vaccination in association with parenteral antioxidant supplementation at day 0 of life in calves. Newborn calves experience elevated oxidative stress risk throughout the first weeks of life [9,10,11], and our previous in vitro data demonstrated compromised adaptive immune responses due to oxidative stress in newborn dairy calves [12]. Similarly, antioxidant-supplemented dogs showed decreased DNA damage and higher vaccine-specific virus-neutralizing antibody levels at 2, 4, and 6 weeks postvaccination than unsupplemented controls [35]. Therefore, the improved antioxidant potential observed throughout the first month of life in the calves that received either Supplement A or Supplement B compared to the control might explain the improved IgA response observed in supplemented calves, given the known direct link between oxidative stress and the immune system in cattle and other species [11].

The sample size utilized in this proof-of-principle study was limited, and we just monitored the effect of parenteral antioxidant supplementation throughout the first month of life because this is the period where newborn calves have been reported to show oxidative stress [9] and the period when most calves succumb to respiratory disease in the herd of the study [36]. Although we were able to demonstrate a positive effect of both commercially available supplements on antioxidant potential and intranasal vaccine response during this period, future multi-herd studies employing larger sample sizes and longer study times are required to investigate the duration of the positive effects reported in this study and the extent to which this results in a decrease in neonatal disease incidence. Furthermore, the vaccine used in this study is licensed for use in cattle 1 week of age or older, and 1-week-old calves are expected to have a more mature immune system than newborn calves [5]. Thus, the effect of antioxidant supplementation on the response to an intranasal vaccine administered according to the label remains unexplored. However, extra-label administration of intranasal vaccines at birth is routine practice at most dairy farms in the USA [6]. Therefore, the results of our study are representative of how these vaccines are used in the dairy industry.

## 4. Conclusions

The administration of parenteral antioxidant supplements simultaneously with an intranasal viral vaccine to newborn calves resulted in an increased response to the vaccine as assessed by antigen-specific immunoglobulin A concentrations in nasal secretions throughout the first month of life. Thus, antioxidant supplementation of newborn calves as undertaken in this study could be a practical strategy to optimize calves’ immunity.

## Figures and Tables

**Table 1:** Distribution of sex and median (range) values of birth weight and serum total protein at 1 week of age across treatment groups.

Variable	Control	Supplement A	Supplement B	*p*-Value
Sex (*n* female/*n* male)	6 ^a^/1	6 ^a^/1	6/1	1.0
Birth weight (kg)	37.2 (29.0–51.7)	36.7 (29.9–43.5)	37.2 (32.2–47.2)	0.98
Serum total protein (g/dL)	6.5 (6.3–6.8)	6.6 (6.2–6.9)	6.5 (6.2–6.8)	0.93

^a^ One heifer calf in each of these groups died after the week 1 sample collection. Results were compared statistically with Fisher’s exact test (sex) or Kruskal–Wallis test (birth weight and serum total protein).

**Table 2:** Model main effect estimates of treatments for the outcome variables of the study. Results are presented as least squared means and 95% confidence intervals.

Variable (*Units*)	Treatment Groups			*p*-Values		
	Control	Suppl. A	Suppl. B	Control vs. Suppl. A	Control vs. Suppl. B	Suppl. A vs. Suppl. B
Reactive Oxygen and Nitrogen Species (*RFU*)	15.6 (14.5–16.7)	11.7 (10.6–12.9)	11.3 (10.2–12.5)	0.0003	0.0001	0.85
Antioxidant Potential (*TE/μL*)	5.09 (3.85–6.33)	8.07 (6.82–9.32)	7.46 (6.21–8.71)	0.0062	0.0288	0.76
Oxidant Status index (*arbitrary units*)	3.23 (2.82–3.65)	1.78 (1.35–2.22)	1.88 (1.45–2.32)	0.0002	0.0005	0.94
Anti-BHV1 IgA (*log (OD_450_)*)	−2.27 (−2.39–−2.15)	−1.63 (−1.76–−1.50)	−1.65 (−1.79–−1.53)	<0.0001	<0.0001	0.93
Anti-BRSV IgA (*log (OD_450_)*)	−2.43 (−3.10–−1.76)	−1.12 (−1.77–−0.67)	−1.44 (−2.09–−0.79)	0.036	0.024	0.74

Suppl. = Supplement; RFU = Relative Fluorescence Units; TE = Trolox equivalents; OD450 = Optical density at 450 nm. Results were analyzed using mixed models with repeated measures, including the main effects of treatment (control, supplement A, or supplement B), time (weeks of age), and the treatment x time interaction (Trt x T). Tukey’s honest significance test was used for pairwise comparisons.

## Data Availability

All data generated or analyzed during this study are included in this published article.
